# Editorial: Advances in the Development of Artificial Metalloenzymes

**DOI:** 10.3389/fchem.2019.00599

**Published:** 2019-08-28

**Authors:** Tatjana N. Parac-Vogt, Andrea Erxleben, Gerhard Schenk, Rajeev Prabhakar

**Affiliations:** ^1^Department of Chemistry, KU Leuven, Leuven, Belgium; ^2^School of Chemistry, National University of Ireland, Galway, Ireland; ^3^School of Chemistry and Molecular Biosciences, The University of Queensland, St. Lucia, QLD, Australia; ^4^Department of Chemistry, University of Miami, Coral Gables, FL, United States

**Keywords:** metalloenzymes, catalysis, active sites, reactions, mechanisms

A wide range of chemical reactions such as hydrolyses, oxidations/reductions, isomerizations, and ligations are catalyzed by metalloenzymes. Many of these reactions have great potential for applications in the biotechnology and pharmaceutical industries. However, only a few of these enzymes have yet been used in such applications, frequently with some shortcomings (e.g., low stability, non-optimal substrate specificity, and high production costs). Therefore, in the last few decades intensive efforts have been made for the design of efficient, selective, environmentally friendly, and economical synthetic analogs of metalloenzymes, also called artificial metalloenzymes. As a result, multiple types of such analogs have been developed, including supramolecular, polymeric, nanoparticulate and low-molecular-weight organometallic complexes, and metal organic frameworks. In this Research Topic, we present a collection of original research and review articles that show significant recent advances made in the rational design of artificial metalloenzymes.

Metal complexes have been commonly used for the hydrolysis of extremely stable peptide and phosphoester bonds. Anyushin et al. demonstrated that Hf(IV)-substituted Wells-Dawson polyoxometalate (POM), K_16_[Hf(α_2_-P_2_W_17_O_61_)_2_] (Hf1-WD2) can act as an efficient and site-selective artificial protease by hydrolyzing ovalbumin (OVA). They proposed that the positively charged patches on the surface of OVA were critical for its activity rather than its overall charge. Crans et al. showed that a Wells-Dawson POM cluster [P_2_W_18_O_62_]^6−^ interacts with the ribosome through the W=O sites of the POM and stabilize its structure. This complex will help crystallographers to address the phase problem and improve a high-resolution X-ray structure of the ribosome. Horn et al. synthesized and characterized binuclear analogs of Ni-containing phosphoesterase enzymes [Ni complex of 1,3-bis(bis(pyridin-2-ylmethyl)amino)propan-2 ol]. An outstanding question concerning the mechanism of dimetallic phosphoesterases is the identity of the nucleophile (bridging or terminal water). They showed that this complex utilizes a terminal water molecule, rather than a bridging one, as a nucleophile. Hu et al. employed Density Functional Theory (DFT) calculations to investigate the phosphatase activities of six di- and tetravalent metal-cyclen (M-C) complexes (Zn-C, Cu-C, Co-C, Ce-C, Zr-C, and Ti-C). They showed that activities of these complexes can be rationally predicted using the metal-ligand and metal-nucleophile bond lengths, strain of the cyclen ring, atomic charges, and coordination number of the metal ion as parameters. Despite the remarkable success of metal complexes in unraveling the mechanisms of hydrolytic reactions, this approach has not been without challenges. Erxleben reviewed the current mechanistic understanding that has been gained from these molecules as well as the limitations of this strategy. The importance of the often overlooked regeneration of these hydrolyzing agents was also highlighted. In another review on this topic, Williams and Grant discussed the progress in hydrolysis of lipids by a wide range of di- and tetra-valent metal ion centers. In particular, the roles of conditions such as temperature and pH in their activities were explored.

In addition to hydrolysis, metal complexes have been successfully employed to study oxidation and decarboxylation reactions. Leone et al. designed an Mn-containing artificial enzyme belonging to the mimochrome VI (MC6) family with peroxygenase activity. Quite interestingly, it served as a bridge between native proteins and small-molecule catalysts. Das et al. synthesized and characterized a new Fe-oxo cluster with a sulfur containing ligand for alkene oxidation. This cluster catalyzed the reaction partially through a metal-centered process in tandem with free radical oxidation by reactive oxygen species. On the other hand, Costa et al. reported the synthesis and characterization of an unusual binuclear mixed-valent Mn-Mn compound with dual catalase and superoxide dismutase activities. The often elusive intermediates in both reactions were also identified. Ahmadi et al. designed a Mo^IV^ mono-oxido bis-dithiolene complex, [MoO(mohdt)_2_]^2−^ (mohdt = 1-methoxy-1-oxo-4-hydroxy-but-2-ene-2,3-bis-thiolate) as a model for molybdenum oxidoreductase enzymes to catalyze an oxygen atom transfer reaction. This aliphatic ligand-containing complex was shown to exhibit an activity comparable to that of its counterparts that contain aromatic dithiolene ligands. Sheng et al. employed combined theoretical and experimental techniques to study the mechanism of C-C bond cleavage by the metal-dependent iso-orotate decarboxylase (IDCase). They also predicted the identity of the relevant metal ion (Mn or Zn) and the substrate specificity of IDCase. Finally, Singh reviewed the enormous potential of nanozymes in biomedical applications. The advantages and disadvantages of nanozymes over natural, as well as artificial enzymes were discussed.

The rational design of efficient artificial metalloenzymes has been widely acknowledged as one of the holy grails in chemistry. However, in the last few decades impressive advancements have been made in the design and synthesis of molecules to mimic both structural and functional aspects of natural metalloenzymes. Since the existing synthetic analogs of enzymes are still quite inferior in terms of rate, selectivity and turnover, we believe that it will continue to be an exceedingly important area of research.

## Author Contributions

All authors listed have made a substantial, direct and intellectual contribution to the work, and approved it for publication.

### Conflict of Interest Statement

The authors declare that the research was conducted in the absence of any commercial or financial relationships that could be construed as a potential conflict of interest.

